# Adding gene transcripts into genomic prediction improves accuracy and reveals sampling time dependence

**DOI:** 10.1093/g3journal/jkac258

**Published:** 2022-09-26

**Authors:** Bruno C Perez, Marco C A M Bink, Karen L Svenson, Gary A Churchill, Mario P L Calus

**Affiliations:** Hendrix Genetics B.V., Research and Technology Center (RTC), 5830 AC Boxmeer, The Netherlands; Hendrix Genetics B.V., Research and Technology Center (RTC), 5830 AC Boxmeer, The Netherlands; The Jackson Laboratory, Bar Harbor, ME 04609, USA; The Jackson Laboratory, Bar Harbor, ME 04609, USA; Animal Breeding and Genomics, Wageningen University & Research, 6700 AH Wageningen, The Netherlands

**Keywords:** genomic prediction, gene transcripts, complex traits, outbred mice

## Abstract

Recent developments allowed generating multiple high-quality ‘omics’ data that could increase the predictive performance of genomic prediction for phenotypes and genetic merit in animals and plants. Here, we have assessed the performance of parametric and nonparametric models that leverage transcriptomics in genomic prediction for 13 complex traits recorded in 478 animals from an outbred mouse population. Parametric models were implemented using the best linear unbiased prediction, while nonparametric models were implemented using the gradient boosting machine algorithm. We also propose a new model named GTCBLUP that aims to remove between-omics-layer covariance from predictors, whereas its counterpart GTBLUP does not do that. While gradient boosting machine models captured more phenotypic variation, their predictive performance did not exceed the best linear unbiased prediction models for most traits. Models leveraging gene transcripts captured higher proportions of the phenotypic variance for almost all traits when these were measured closer to the moment of measuring gene transcripts in the liver. In most cases, the combination of layers was not able to outperform the best single-omics models to predict phenotypes. Using only gene transcripts, the gradient boosting machine model was able to outperform best linear unbiased prediction for most traits except body weight, but the same pattern was not observed when using both single nucleotide polymorphism genotypes and gene transcripts. Although the GTCBLUP model was not able to produce the most accurate phenotypic predictions, it showed the highest accuracies for breeding values for 9 out of 13 traits. We recommend using the GTBLUP model for prediction of phenotypes and using the GTCBLUP for prediction of breeding values.

## Introduction

Predicting complex traits is a fundamental aim of quantitative genetics. The use of whole-genome single nucleotide polymorphisms (SNPs) considerably improved the prediction of breeding values, resulting in the process widely known as genomic prediction (Meuwissen *et al.* 2001). A number of statistical approaches are now applied routinely in breeding programs, such as genomic best linear unbiased prediction (GBLUP) (VanRaden 2008), ridge regression (Whittaker *et al.* 2000), or methods from the “Bayesian Alphabet” (Gianola *et al.* 2009). More recently, machine learning algorithms have been tested in the context of genomic prediction (González-Recio *et al.* 2013; Pook *et al.* 2020; Zingaretti *et al.* 2020). These models may have several advantages when compared with the linear models routinely used in animal breeding programs, such as capturing interactions between predictors (nonadditive effects), automatic variable selection, and for making fewer assumptions regarding the underlying genetic architecture of phenotypes (Nayeri *et al.* 2019; Pérez-Enciso and Zingaretti 2019). However, compared to the routinely used models, prediction performance from machine learning methods has shown mixed results (Azodi *et al.* 2019; Abdollahi-Arpanahi *et al.* 2020; Perez *et al.* 2022). There seems to be no “one-size-fits-all” model, as results are dependent on trait genetic architecture, size of the data, and fine-tuning of hyperparameters.

Recent development of low-cost high-throughput molecular technologies allowed multiple high-quality ‘omics’ data to be measured with high accuracy (Fernie and Schauer 2009; Tohge and Fernie 2015; Chawade *et al.* 2016). This has led to interest in utilizing these as new layers of information to improve the predictive performance of genomic prediction models, ultimately contributing to improve efficiency of breeding programs (Guo *et al.* 2016; Li *et al.* 2019). For example, gene expression levels measured in tissue samples by direct RNA sequencing (RNA-seq) are now readily available to animal and plant breeders (Stark *et al.* 2019). To incorporate these new sources of information into genomic prediction models requires new strategies for integration with the already widely used genome-wide marker data. Although most of the literature focusing on the inclusion of gene-expression data into genomic models to improve predictive performance aimed at predicting phenotypes (Takagi *et al.* 2014; Guo *et al.* 2016; Schrag *et al.* 2018; Azodi *et al.* 2019; Li *et al.* 2019; Morgante *et al.* 2020), fitting gene transcript levels as an additional layer of information into genomic models could indirectly improve the prediction of breeding values. Christensen *et al.* (2021) presented a 2-step method to incorporate such intermediate omics into genomic evaluations considering complete and incomplete omics-data scenarios. Results were validated using simulated data and suggested superiority of the single-step method including both the intermediate omics and genomics data over the traditional GBLUP using only genomics data. Similar results were observed by Michel *et al.* (2021) when investigating the integration of gene expression into genomic prediction for disease resistance in wheat by using a hybrid relationship matrix for merging both layers of omics data.

After observing much less variation in the gene expression among monozygotic twins than among siblings or unrelated individuals, Cheung *et al.* (2003) suggested a strong association between genotypes and the level of gene expression in humans. This finding is an indication that SNP genotypes and gene transcripts might contain redundant information which turns into a challenge when these are used as predictors in genomic models. Therefore, an adequate handling of these associations is necessary to prevent inflated relative contributions of individual layers (Holm *et al.* 2010; Christensen *et al.* 2021). In the same line of thought, Wade *et al.* (2022) have emphasized that the benefits of multiomics integration models over single-omic models are achieved once redundancy of predictors is decreased. Consequently, multiomics models should either automatically or through adequate parametrization be able to identify and manage information redundancy across multiple omics layers.

In this study, we used data from the Diversity Outbred (DO) mouse population (Churchill *et al.* 2012; Svenson *et al.* 2012) to evaluate the utility of gene expression in addition to genome-wide genetic markers for genomic prediction using different modeling strategies. To this end, the objectives of this study were to: (1) estimate the proportions of phenotypic variance explained by genetic markers and gene transcripts in complex traits recorded in at least 2 time points to assess sampling time dependency; (2) evaluate the predictive accuracy for phenotypes using transcripts and/or marker information for the traits investigated using linear models and machine learning approaches; and (3) evaluate how the inclusion of transcripts affects estimation of genomic breeding values (GEBV) from linear models. We considered best linear unbiased prediction (BLUP) as a representative of linear models, while the gradient boosting algorithm was used as the machine learning representative. The BLUP models tested here vary in number of components, how interactions were modeled, and conditioning of one component on another. In addition to more traditionally used models, we developed and tested a new model called GTCBLUP with the objective to reduce redundancy between genomics and transcriptomics layers. The gradient boosting algorithm is a nonparametric machine learning method and was chosen for its ability to automatically control redundancy and implicitly account for nonlinear effects in prediction, while the BLUP models tested comprise parametric approaches to incorporate genomics and transcriptomics, considering or ignoring the interactions between them.

## Materials and methods

### Data

#### Phenotypes

Data used for this study were obtained from The Jackson Laboratory (Bar Harbor, ME, USA) and comprise a subset of the dataset used in Perez *et al.* (2022). The 478 DO mice originated from 4 nonoverlapping generations (4, 5, 7, and 11) with males and females represented equally. The total number of animals per generation was 47, 47, 192, and 192 for generations 4, 5, 7, and 11, respectively, with slight variation in the numbers of missing records across traits (Table 1). The mice were maintained on either standard high-fiber (chow, *n* = 239) or high-fat diet (*n* = 239) from weaning until 23 weeks of age. The proportion of males and females within each diet category was close to 50–50 for all generations, as well as within each litter-generation combination (2 litters per generation). This population is maintained under a systematic mating scheme, designed to limit population structure and relatedness. On average, the animals were related to each other at a level equivalent to first cousins, which is by design (Svenson *et al.* 2012). More elaborate description of population structure, husbandry and phenotyping methods can be found in Svenson *et al.* (2012) and Tyler *et al.* (2021).

**Table 1.** Number of available observations (*N*), the extended description of traits, age of the animals at phenotype measurement, and estimated heritability.

Trait	*N*	Trait description	Age at measurement (wk)	**Estimated heritability** ^*a*^
BMD12	471	Bone mineral density	12	0.39
BMD21	471	Bone mineral density	21	0.41
BW10	478	Body weight	10	0.42
BW15	478	Body weight	15	0.35
BW20	478	Body weight	20	0.37
CHOL8	474	Circulating cholesterol	8	0.38
CHOL19	474	Circulating cholesterol	19	0.45
FATP12	471	Body fat percentage	12	0.35
FATP21	471	Body fat percentage	21	0.32
GLUC8	425	Circulating glucose	8	0.31
GLUC19	425	Circulating glucose	19	0.22
TRGL8	473	Circulating triglycerides	8	0.36
TRGL19	473	Circulating triglycerides	19	0.31

a Standard errors for the heritability ranged from 0.07 to 0.09.

Table 1 gives for each trait a brief description, the numbers of observations, and the estimated heritability. We considered 6 traits based on range of heritability and presumed genetic architectures. The chosen traits were measured 2 or 3 times during the animal’s life, resulting in 13 distinct traits in total. The analyzed traits were bone mineral density at 12 (BMD12) and 21 (BMD21) weeks; body weight at 10, 15, and 20 weeks (BW10, BW15, and BW20); circulating cholesterol at 8 (CHOL8) and 19 (CHOL19) weeks; adjusted body fat percentage at 12 (FATP12) and 21 (FATP21); circulating glucose at 8 (GLUC8) and 19 (GLUC19) weeks; and circulating triglycerides at 8 (TRGL8) and 19 weeks (TRGL19). These traits can be categorized into measurements of body composition (bone mineral density, body weights, and fat percentage) and clinical plasma chemistries (circulating glucose and triglycerides). To allow a fair comparison between parametric and nonparametric models compared in this study, phenotypic records were precorrected for fixed effects of diet, generation, litter, and sex (Perez *et al.* 2022). Therefore, the precorrected phenotypes ($y^{*})$ analyzed here comprise the sum of the additive genetic effect and residual terms.

#### Genotypes

The genotype data used for the animals in this study were obtained from their derived founder haplotypes (for details see Perez *et al.* 2022). The complete genotype file used for the analyses included 64,000 markers on an evenly spaced grid, and the average distance between markers was 0.0238 cM. The full genotype dataset was cleaned based on the following criteria: variants with minor allele frequency <0.05, call rates <0.90, and linear correlation between subsequent SNPs >0.80 were removed. After quality control, a total of 50,122 SNP markers were available for the mice with phenotypic, genotypic, and transcriptomic records.

#### Transcript levels

Transcriptome-wide expression levels were measured from whole livers as previously described (Munger *et al.* 2014; Chick *et al.* 2016) for 478 animals at 26 weeks of age. The RNA sample was sequenced using single-end RNA-Seq (Munger *et al.* 2014) and aligned transcripts to strain-specific genomes from the DO founders (Chick *et al.* 2016). Read counts were estimated using an expectation–maximization algorithm (EMASE, https://github.com/churchill-lab/emase). Read counts were previously corrected for the effects of sex, diet, and batch by normalizing in each sample using upper quantile normalization and applying a rank Z transformation across samples. After quality control, quantification of transcripts was available for 11,770 genes (Tyler *et al.* 2017).

### Best linear unbiased prediction

#### GBLUP

Below we introduce 5 BLUP models and 3 gradient boosting machine (GBM) models with their acronyms and key features summarized in Table 2. The statistical model of GBLUP is:

y*=1μ+g+e,

where y* is the vector of precorrected phenotypes, **1** is a vector of ones, μ is the intercept, and g is the vector of random additive genetic values, where $g\;∼\;N(0,\;G\sigma_{g}^{2})$, G is the additive genomic relationship matrix between genotyped individuals, and $\sigma_{g}^{2}$ is the additive genomic variance. The matrix G is constructed following the second method described by VanRaden (2008) as $\frac{{\mathrm{ZZ}}^{\prime }}{m}$, where Z is the matrix of centered and standardized genotypes for all individuals and m is the number of markers. Finally, e is the vector of random residual effects, where $e\;∼\;N(0,\;{I\sigma }_{e}^{2})$ with $\sigma_{e}^{2}$ being the residual variance, and I is an identity matrix.

**Table 2.** Overview of models applied to SNP genotypes and/or individual levels of gene transcripts.

Model acronym		Explanatory variables		
		SNP genotypes	Gene transcripts	Interaction modeled
GBLUP		Yes	No	No
	GGBM	Yes	No	Yes (implicitly)
TBLUP		No	Yes	No
	TGBM	No	Yes	Yes (implicitly)
GTBLUP		Yes	Yes	No
GTCBLUP		Yes	Yes	No
GTIBLUP		Yes	Yes	Yes (explicitly)
	GTGBM	Yes	Yes	Yes (implicitly)

#### TBLUP

To evaluate the performance of transcriptomic data for predicting complex traits, we used a Transcriptomic Best Linear Unbiased Predictor (TBLUP) model. This model is similar to GBLUP, but using a transcriptomic relationship matrix, which evaluates the similarity among animals based on gene expression levels (Guo *et al.* 2016).

The statistical model of TBLUP is:

y*=1μ+t+e,

where y*, **1**, and μ are defined as above, t is the vector of random transcript level effects, where $t\;∼\;N(0,\;T\sigma_{t}^{2})$ and T is the transcriptomic relationship matrix built according to the formula $\frac{{\mathrm{WW}}^{\prime }}{k}$, where W is the matrix of centered and standardized expression levels for all animals and *k* is the number of genes, and $\sigma_{t}^{2}$ is the variance explained by gene transcripts.

#### GTBLUP and GTIBLUP

The GTBLUP model fitted the g and t as independent random effects, each with their own variance component (Guo *et al.* 2016; Li *et al.* 2019). The model is y*=1μ+g+t+e, where all the parameters are as defined above.

The GTIBLUP model fitted g, t, and the interaction between g and t with an additional variance component (Morgante *et al.* 2020). This model is y*=1μ+g+t+gt+e, where y*, 1μ, g, t, and e are as defined above, and gt is the vector of interaction (between genomic and transcriptomic) effects, where $\mathrm{gt}\;∼\;N(0,\;{G\#T\sigma }_{gt}^{2})$ and # is the Hadamard product.

#### GTCBLUP

The newly developed GTCBLUP model was similar to GTBLUP in that the g and t that were fitted as independent random effects, each with their own variance component. However, for this model, the transcript levels were conditioned on SNP genotypes, yielding a matrix $W_{c}$ computed as: $W_{c}=(I-{Z\left(Z^{\prime }Z+I\lambda \right)}^{-1}Z^{\prime })W$, where ${Z\left(Z^{\prime }Z+I\lambda \right)}^{-1}Z^{\prime }$ is the so-called “smoother matrix” (Hastie *et al.* 2009), Z is the matrix of centered and standardized genotypes as before, I is an identity matrix, and $\lambda =\frac{m\;*\;\sigma_{e}^{2}}{\sigma_{g}^{2}}$, $\sigma_{e}^{2}$ is the residual variance, and $\sigma_{g}^{2}$ is the additive genomic variance, both variances estimated with the GBLUP model (including only g). Using the smoother matrix, i.e. including Iλ rather than using $I-{Z\left(Z^{\prime }Z\right)}^{-1}Z^{\prime }$, reflects that the effects associated with the SNPs are estimated as random rather than fixed effects. The aim of this model is to remove any variance from transcripts that is correlated to variance in genotypes, such that any phenotypic variance both associated with variance in genotypes and transcripts automatically will be associated with the genotypes only. Our hypothesis is that when using the GTCBLUP model for genomic prediction, any overlapping information contained between SNP genotypes and gene transcripts is removed, allowing the model to perform a more accurate partition of variance for the genetic component, and ultimately, increase prediction accuracy for GEBV. The model is $y*=1\mu +g+t_{c}+e,$ where $t_{c}\;∼\;N(0,\;T_{c}\sigma_{t}^{2})$ and $T_{c}$ is computed as $\frac{W_{c}W_{c}^{\prime }}{k}$, and all other parameters are as defined above.

### Gradient boosting machine models

GBM is an ensemble learning technique that applies an iterative process of assembling “weak learners” into a stronger learner, being largely used for both classification and regression problems (Friedman 2001). In the scope of this investigation, the GBM algorithm represents a nonparametric approach capable of implicitly fitting not only the additive effects of SNP and gene transcripts but also the within- and between-omics layers interactions. The GBM is also capable of performing automatic feature selection, prioritization of important variables, and discarding variables containing irrelevant or redundant information. A detailed description of the GBM algorithm and its application in genomic prediction can be found in Friedman (2002), González-Recio *et al.* (2010, 2013), and Perez *et al.*, (2022).

To obtain the best possible results from the GBM algorithm, a grid search approach was used to determine the combination of hyperparameters that minimized the mean squared error of prediction within the inner training set for each trait. Details of the hyperparameter search method used are found in Perez *et al.* (2022). We implemented the GBM model using the “gbm” R package (Ridgeway 2020).

We tested 3 different GBM models. The first model considered only SNP genotypes as predictors (GGBM), the second model considered only (standardized) gene transcript levels as predictors (TGBM), and a third model that considered both genetic markers and transcript levels together as predictors (GTGBM). Our objective was to investigate if GBM models could capture within- and between-omics layers associations, while also reducing within- and between-omics layers redundancy by performing automatic variable selection. It is important to note here that although here we used “G” and “T” letters to refer to genomics and transcriptomics data in the GBM model’s acronyms, predictors were fit directly in the model and not as relationship matrices.

### Variance explained by genetic markers, transcript levels, and combinations of both

To understand how much of the phenotypic variance can be explained by using SNP genotypes, gene transcript levels and the combinations of both sources of information, we estimated variance components using the GBLUP, TBLUP, GTBLUP, GTIBLUP, and GTCBLUP models. Estimates of variance components along with the residual variance ($\sigma_{e}^{2}$) were obtained from a Bayesian approach analysis, using the BGLR R package (Pérez and de los Campos 2014). The residual variance and variances from random effects included in the models were assigned a scaled-inverse $\chi^{2}$ density $p\left(\sigma_{\theta }^{2}\right)=\chi^{-2}(\sigma_{\theta }^{2}|S_{\theta },{df}_{\theta })$, where θ represent the variance component ($g,t,t_{c},gt,$ or e), S and df are the scale and degree of freedom parameters. The value of 5 was used for df in all models. The parameter S was calculated for $\sigma_{e}^{2}$ as $S_{e}=\sigma_{p}^{2}*\;{(1-R}^{2})*\left({df}_{e}-2\right)$, where $\sigma_{p}^{2}$ is the phenotypic variance and $R^{2}$ is the prior expectation for the proportion of variance to be explained by the model. For all other variance components, S was calculated as $S_{\theta }=\sigma_{p}^{2}*\;R^{2}*\frac{\left({df}_{\theta }+2\right)}{\mathrm{mean}\left(\mathrm{diag}\left(K\right)\right)}$, where K represents the relationship matrix assigned to the respective variance component (θ). For all models, the Gibbs sampler was run for 60,000 iterations, with a 20,000 burn-in period and a thinning interval of 10 iterations. Consequently, inference was based on 4,000 posterior samples.

For the GTIBLUP model, we calculated the proportion of variance explained by SNP genotypes ($h^{2}=\frac{\sigma_{g}^{2}}{{\sigma_{g}^{2}+\sigma }_{t}^{2}+\sigma_{gt}^{2}+\sigma_{e}^{2}})$, gene transcripts ($t^{2}=\frac{\sigma_{t}^{2}}{{\sigma_{g}^{2}+\sigma }_{t}^{2}+\sigma_{gt}^{2}+\sigma_{e}^{2}}$), and from the interaction between effects from genetic markers and gene transcripts (${gt}^{2}=\frac{\sigma_{gt}^{2}}{{\sigma_{g}^{2}+\sigma }_{t}^{2}+\sigma_{gt}^{2}+\sigma_{e}^{2}}$). Consequently, the sum of $h^{2}$, $t^{2},$ and ${gt}^{2}$ represent the proportion of the phenotypic variance explained by 2 layers of omics data ($h^{2}$ and $t^{2})$ and by the between-omics-layer interactions (${gt}^{2})$. The parameters $h^{2}$, $t^{2},$ and ${gt}^{2}$ were calculated similarly for the other models but omitted any variance components associated with effects not included in the model.

### Predictive performance for phenotypes

Performance of predictions from the models was measured by the accuracy, computed as the Pearson correlation ($r_{y^{*},\hat{y}}$), and the relative root-mean-squared error of prediction (RRMSE) between predictions ($\hat{y})$ and precorrected phenotypes ${(y}^{*})$: RRMSE = $\sqrt{\frac{1}{n}\;\sum_{i=1}^{n}({y^{*}-\hat{y})}^{2}}/\sigma_{p}$, where $\sigma_{p}$ is the phenotypic standard deviation. In all analyses, we used a forward prediction validation scheme in which animals from older generations (4, 5, 7) were used as the reference and animals from the younger generation (11) as the validation subset. The standard error (SE) around the $r_{y^{*},\hat{y}}$ estimates was obtained by calculating the standard deviations from 10,000 bootstrap samples (Davison and Hinkley 1997). The bootstrapping procedure was implemented using the “boot” R package (Canty and Ripley 2021). Statistical difference between prediction accuracies from different models was tested using the Hotelling–Williams test for dependent correlations (Steiger 1980). We have also assessed prediction bias by obtaining the regression coefficient from the linear regression of corrected phenotypes on model predictions. For these results, values above 1 indicate deflation, while values below 1 indicate inflation of predicted values.

To assess the proportion of variance explained by the models tested, we have calculated the coefficient of determination (*R*^2^) from the regression of corrected phenotypes on model predictions for all traits. For the GBM models, we have used results from the model using the previously obtained best hyperparameter set from the standard grid-search procedure to assess the model *R*^2^ for prediction within the reference set.

### Predictive performance of different model components

For the BLUP models proposed to integrate SNP genotypes and gene transcripts (GTBLUP, GTIBLUP, and GTCBLUP), in addition to $r_{y^{*},\hat{y}}$ we have also calculated the correlations between the solutions for each random effect included in the model (g, t, $t_{c},$ or gt) and $\hat{y}$, as well as pairwise comparisons between all components in the model. Noting that the model component g in fact are GEBV, also the predictive performance for GEBV for any of the models was evaluated as the Pearson correlation between g and $\hat{y}$. We did not divide those by the square root of the heritability, which would transform them into accuracies of GEBV with an interpretation of being the correlation between estimated and true breeding values, but instead evaluated the predictive performance of each model component in terms of the accuracy of predicting phenotypes. This enabled direct comparison across the different model components. Evaluating solutions from the additive genetic component from these models enabled to assess if the prediction of GEBV can be improved by using models capable of integrating SNP genotypes and gene transcripts for genomic prediction.

## Results

### Variance components estimation percentage of variance explained within the reference set

**Fig. 1.**
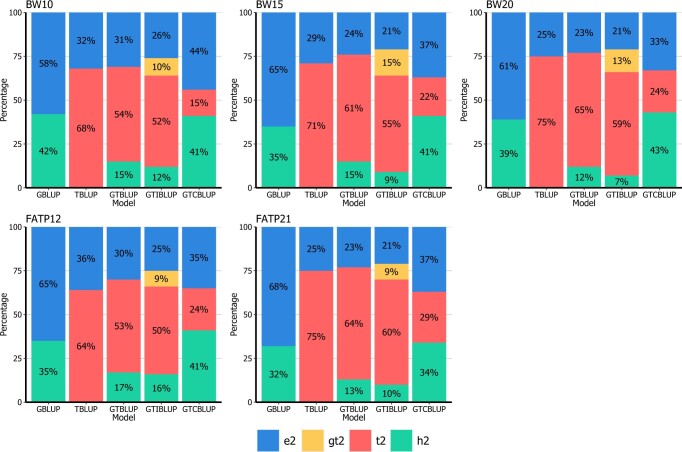
Percentage of variance explained by SNP genotypes ($g^{2}$), gene transcripts ($t^{2}$), the interaction between them (${gt}^{2}$), and not explained ($e^{2}$) by GBLUP, TBLUP, GTBLUP, GTIBLUP, and GTCBLUP models tested for the traits BW and FATP. For a description of the traits, see Table 1. For a description of the models, see Table 2.

**Fig. 2.**
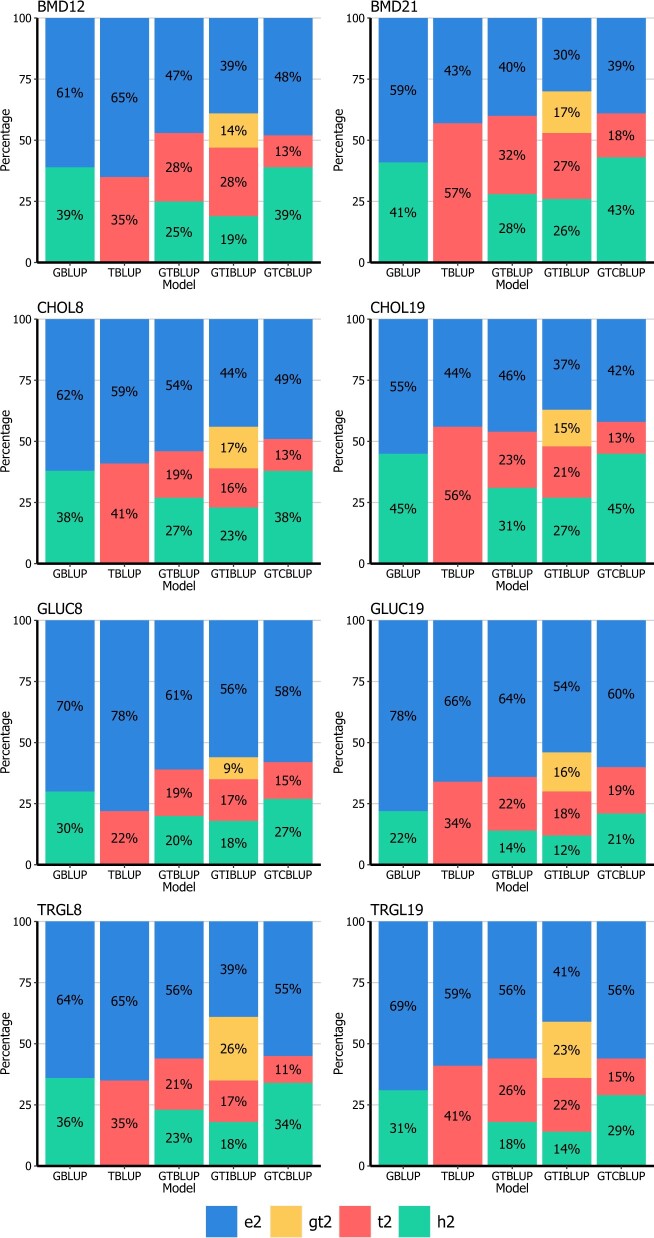
Percentage of variance explained by SNP genotypes ($g^{2}$), gene transcripts ($t^{2}$), the interaction between them (${gt}^{2}$), and not explained ($e^{2}$) by GBLUP, TBLUP, GTBLUP, GTIBLUP, and GTCBLUP models tested for the traits BMD, CHOL, GLUC, and TRGL. For a description of the traits, see Table 1. For a description of the models, see Table 2.

Genomic heritabilities ($h^{2}$) obtained with GBLUP ranged from 0.08 to 0.44, representing a wide range of magnitudes across traits (Figs. 1 and 2). The estimated values for each variance component for all traits is presented in Supplementary Tables 1 and 2. When only fitting transcript levels as predictors (TBLUP), the percentage of variance explained ($t^{2}$) ranged from 0.22 to 0.75 and in general, it was higher than $h^{2}$ when comparing within the same trait. The exceptions to that were observed for BMD12 ($h^{2}$ = 0.39 and $t^{2}$ = 0.35) and GLUC8 ($h^{2}$ = 0.30 and $t^{2}$ = 0.22). When comparing the same trait measured at different time points, $t^{2}$ from TBLUP was higher for phenotypes collected closer to 26 weeks of age (i.e. the age at mRNA data sampling).

In terms of the total phenotypic variance explained, GTBLUP and GTIBLUP showed similar results (Figs. 1 and 2). For body weights (BW10, BW15, and BW20) and fat percentage (FATP12 and FATP21) traits, the variance explained by genetic markers (in GTBLUP and GTIBLUP) was drastically lower when compared with GBLUP for the same traits. For the remaining traits also a decrease in genetic variance captured by markers was observed, albeit that the decrease was much lower. For the interaction component in GTIBLUP (${gt}^{2}$), results observed varied according to the trait analyzed but in general, it was low compared to both $h^{2}$ and $t^{2}$. The only exception to that was observed for TRGL8, in which ${gt}^{2}$ was higher than $h^{2}$ and $t^{2}$. For CHOL8, GLUC19, and TRGL19, ${gt}^{2}$ was either similar to $h^{2}$ or $t^{2}$.

For GTCBLUP, differently from GTBLUP and GTIBLUP, the additive genetic variance captured was always in line with results from GBLUP. On the other hand, the variance explained by transcripts ($t^{2}$) from GTCBLUP was always lower than observed by other models including transcripts as predictors (TBLUP, GTBLUP, and GTIBLUP).

The variance explained (represented by the *R*^2^ parameter) within the reference data by parametric models was in general lower than by the nonparametric counterparts (Table 3). Independent of being a parametric or nonparametric model, the use of gene transcripts (TBLUP and TGBM) as predictors explained in most cases more of the variance than using exclusively SNP genotypes (GBLUP and GGBM). For GTBLUP, GTIBLUP, and GTGBM, the variance explained was at least similar to observed for TBLUP and TGBM, but generally higher. For GTCBLUP, variance explained by the model was slightly to moderately higher than observed for GBLUP model, but always smaller than observed for GTBLUP, GTIBLUP, and GTGBM. The average *R*^2^ when considering only traits recorded earlier (suffixes 8, 10, or 12) and later (suffixes 19, 20, or 21) moments were 76% and 83%, respectively, when using TBLUP, being the largest difference observed across models when considering these 2 groups of traits.

**Table 3.** Model *R*^2^ (×100) for the best linear unbiased prediction (GBLUP, GTBLUP, GTCBLUP, and GTIBLUP) and gradient boosting (GGBM, TGBM, and GTGBM) approaches within training data.

**Trait** *^a^*	**Model** *^b^*							
	Only SNP		Only gene transcripts		SNP + gene transcripts			
	GBLUP	GGBM	TBLUP	TGBM	GTBLUP	GTIBLUP	GTCBLUP	GTGBM
BMD12	75	90	79	96	85	92	88	95
BMD21	84	85	88	93	87	96	92	98
BW10	78	92	93	96	91	95	90	97
BW15	75	87	91	94	93	96	94	96
BW20	80	87	92	93	95	97	95	97
CHOL8	84	85	69	97	80	93	87	98
CHOL19	82	83	85	95	88	96	92	98
FATP12	75	76	89	97	92	96	95	98
FATP21	80	82	93	97	95	97	94	98
GLUC8	77	85	66	95	81	93	87	97
GLUC19	62	80	67	96	75	90	84	98
TRGL8	65	81	62	92	80	96	88	97
TRGL19	71	86	70	96	78	95	84	98
Mean (all)	76	85	80	95	86	95	90	97
Mean (T1)*^c^*	75	85	76	96	85	94	89	97
Mean (T2)*^c^*	76	84	83	95	86	95	90	98

T1 = average *R*^2^ of the column considering only traits recorded earlier in life (suffixes 8, 10, and 12).

T2 = average *R*^2^ of the column considering only traits recorded later in life (suffixes 19, 20, and 21).

a For a description of the traits, see Table 1.

b For a description of the models, see Table 2.

c BW15 trait was ignored when calculating average performance considering exclusively T1 and T2.

### Prediction performance—phenotype prediction

In Table 4, accuracies are shown for predicted phenotypes for BLUP and GBM models using either SNP genotypes, transcript levels or both as predictors. Corresponding standard errors are presented in Supplementary Table 3. Here, we considered GBLUP to be the reference method. It showed prediction accuracies ranging from 0.01 to 0.29, these were highly positively correlated to the portion of variance explained by SNP genotypes by the same model, except for CHOL19. When comparing predictive performance between GBLUP and GGBM models, GBLUP yielded the highest prediction accuracies for 7 traits, while GGBM had the best predictive performance for 6 traits out of 13.

**Table 4.** Accuracies of predicted precorrected phenotypes for the validation subset with the proposed models.

**Trait** *^a^*	**Model** *^b^*							
	Only SNP		Only gene transcripts		SNP + gene transcripts			
	GBLUP	GGBM	TBLUP	TGBM	GTBLUP	GTIBLUP	GTCBLUP	GTGBM
BMD12	**0.19** ^a^	0.16^a^	**0.27** ^b^	0.25^a,b^	0.27 ^b^	0.27 ^b^	0.20^a^	0.25^a,b^
BMD21	**0.29** ^a^	0.28^a^	0.38 ^a,b^	0.38 ^a,b^	**0.42** ^b^	0.40^b^	0.29^a^	0.38^a,b^
BW10	0.20 ^a^	0.20 ^a^	**0.48** ^c^	0.42^b^	0.47 ^c^	0.47 ^c^	0.19^a^	0.42^b^
BW15	**0.17** ^b^	0.12^a^	0.52 ^c^	0.52 ^c^	0.51 ^c^	0.51 ^c^	0.22^b^	0.49^c^
BW20	**0.18** ^a^	0.16^a^	**0.61** ^c^	0.58^c^	0.60 ^c^	0.60 ^c^	0.30^b^	0.54^c^
CHOL8	0.14^a^	**0.17** ^a^	0.14^a^	**0.15** ^a^	**0.18** ^a^	0.16^a^	0.16^a^	0.16^a^
CHOL19	**0.25** ^a,b^	0.20^a,b^	**0.19** ^a,b^	0.16^a^	**0.26** ^c^	0.23^a,b^	0.22^a,b,c^	0.23^a,b,c^
FATP12	**0.16^a^**	0.15^a^	0.44^c^	**0.45** ^c^	0.44^c^	0.44^c^	0.28^a,b^	**0.46** ^c^
FATP21	**0.22** ^a^	0.20^a^	0.54^c^	**0.56** ^c^	**0.54** ^c^	0.53^c^	0.35^b^	0.52^c^
GLUC8	0.10^b,c^	**0.12** ^b,c^	0.03^a^	**0.04** ^a^	0.08^a,b^	0.09^a,b^	0.11^b,c^	**0.15** ^c^
GLUC19	0.01^b^	**0.10** ^c^	0.03^b^	**0.05** ^b,c^	0.04^b^	0.05^b,c^	−0.05^a^	**0.11** ^c^
TRGL8	0.08^a^	**0.11** ^a^	0.06^a^	**0.08** ^a^	0.07 ^a^	0.07 ^a^	0.05^a^	0.06^a^
TRGL19	**0.15** ^a,b^	0.13^a^	0.17^a,b^	**0.19** ^b^	0.17^a,b^	0.18^a,b^	0.12^a^	**0.19** ^b^

For each group of models, the result with the highest accuracy is indicated in bold, identical results between 2 or more models are indicated in underline. For each row, different letters indicate significant differences between models.

a For a description of the traits, see Table 1.

b For a description of the models, see Table 2.

For models that include only gene transcripts (TBLUP and TGBM), the TBLUP approach showed predictive accuracies ranging from 0.03 to 0.61, having the best performance for only 4 out of 13 traits. The TGBM model was able to overcome TBLUP for 7 traits, with prediction accuracies ranging from 0.04 to 0.58. For BMD21 and BW15, predictive accuracy was identical between TBLUP and TGBM. The differences between accuracies from TBLUP and TGBM were higher than between GBLUP and GGBM.

For models that combined SNP genotypes and gene transcripts levels (GTBLUP, GTCBLUP, GTIBLUP, and GTGBM), GTBLUP had the highest predictive accuracy for 5 traits out of 13. The second-best model overall was GTGBM, with the highest predictive accuracy for 4 traits. For every trait that GTIBLUP had the highest prediction accuracy, it was identical to the result for GTBLUP, while the GTCBLUP never had the highest predictive accuracy (Table 4).

The prediction error (RRMSE) and bias (β) for model’s predictions are presented in Supplementary Tables 4 and 5, respectively. Considering single-omics models, on average BLUP models (GBLUP and TBLUP) yielded less biased predictions than GBM models (GGBM and TGBM). For models integrating SNP genotypes and gene transcripts, GTBLUP and GTIBLUP showed similar bias across traits, while GTGBM had on average less bias than the BLUP models. For the GTCBLUP model, predictions were inflated (β<1) for all traits but BMD21. In terms of prediction error, differences between models were smaller than observed for bias (Supplementary Table 5) or predictive accuracies (Table 4). The lowest RRMSE values were observed for FATP21, while the highest RRMSE values were observed for GLUC19. The RRMSE values for all traits analyzed were all around 1, indicating the average prediction errors were close to 1 phenotypic standard deviation.

### Predictive ability for GEBV and other model components, and the correlation between them in BLUP models

In Table 5, the Pearson’s coefficient correlation between model components solutions ($\hat{g}$, $\hat{t}$, $\hat{t_{c}}$, and ${\hat{g}}_{t}$) for the different BLUP models and corrected phenotypes ($y^{*}$) are shown. Corresponding standard errors are presented in Supplementary Table 6. Overall, results for GTBLUP and GTIBLUP were similar across traits. These 2 models had the most accurate GEBV ($\rho_{\hat{g}\_y^{*}}$) exclusively for GLUC8, while for BMD12 results from these models were matched by GTCBLUP. For GLUC19, all 4 parametric multiomics models yielded the same accuracy for GEBV, which was the lowest (0.01) across traits. In 8 out of 13 traits, the GEBV estimated using GTCBLUP model was the most accurate across all models. The correlation between $\hat{t}$ and $y^{*}$ ($\rho_{\hat{t}\_y^{*}}$) was also similar between GTBLUP and GTIBLUP, being always higher for these 2 models than observed for GTCBLUP. For GTCBLUP exclusively, $\rho_{\hat{t}\_y^{*}}$ was low and negative for CHOL19 (−0.08), GLUC19 (−0.05), and TRGL8 (−0.07). For most traits, although a slight increase in the total variance explained was observed within the reference dataset (Figs. 1 and 2) when comparing GTBLUP and GTIBLUP, there was not a proportional increase in $\rho_{\hat{g}\_y^{*}}$ in the validation (Table 5). For GTCBLUP, on the other hand, for all traits, there was an increase in the variance explained by SNP genotypes ($g^{2}$ in Figs. 1 and 2) when compared with GTBLUP and GTIBLUP, and the same pattern was observed for $\rho_{\hat{g}\_y^{*}}$. Results for $\rho_{\hat{g}\_\hat{t}}$ varied from +0.14 to +0.29 for GTBLUP and from +0.13 to +0.29 for GTIBLUP. For GTCBLUP, values for $\rho_{\hat{g}\_\hat{t_{c}}}$ were all negative and close to zero, ranging from −0.13 to −0.03 (Table 5). The values for $\rho_{\hat{g}\_\hat{gt}}$, only calculated for GTIBLUP, were close to zero for most traits with an exception for CHOL8 and CHOL19, for which $\rho_{\hat{g}\_\hat{gt}}$ was 0.18. A similar pattern was observed for $\rho_{\hat{t}\_\hat{gt}}$, for which values varied from −0.12 to +0.06, with the largest differences from $\rho_{\hat{g}\_\hat{gt}}$ observed for CHOL8 and CHOL19.

**Table 5.** Pearson’s coefficient correlation (ρ) between model’s components ($\hat{g}$, $\hat{t}$, $\hat{t_{c}}$, and $\hat{\mathrm{gt}}$) solutions and corrected phenotypes ($y^{*})$ for BLUP models proposed.^*a*^

**Trait** ^*b*^	Model^*c*^											
	GTBLUP			GTIBLUP					GTCBLUP			GBLUP
	$\rho_{\hat{g}\_y^{*}}$	$\rho_{\hat{t}\_y^{*}}$	$\rho_{\hat{g}\_\hat{t}}$	$\rho_{\hat{g}\_y^{*}}$	$\rho_{\hat{t}\_y^{*}}$	$\rho_{\hat{g}\_\hat{t}}$	$\rho_{\hat{g}\_\hat{gt}}$	$\rho_{\hat{t}\_\hat{gt}}$	$\rho_{\hat{g}\_y^{*}}$	$\rho_{\hat{t_{c}}\_y^{*}}$	$\rho_{\hat{g}\_\hat{t_{c}}}$	$\rho_{\hat{g}\_y^{*}}$
BMD12	0.20 ^a^	0.24^u^	0.22	0.20 ^a^	0.22^u^	0.21	−0.04	0.06	0.20 ^a^	0.04^v^	−0.10	0.19^a^
BMD21	0.29^a^	0.38^u^	0.28	0.30^a^	0.37^u^	0.29	0.04	−0.03	**0.31** ^a^	0.08^v^	−0.03	0.29^a^
BW10	0.19^a^	0.46^u^	0.27	0.19^a^	0.47^u^	0.27	0.04	0.02	0.19^a^	0.03^v^	−0.13	**0.20** ^a^
BW15	0.16^a^	0.52^u^	0.29	0.16^a^	0.51^u^	0.28	0.02	−0.02	**0.18** ^a^	0.08^v^	−0.06	0.17^a^
BW20	0.17^a,b^	0.61^u^	0.25	0.17^a,b^	0.60^u^	0.26	−0.09	−0.02	**0.21** ^b^	0.16^v^	−0.10	0.18^a^
CHOL8	0.14^a^	0.13^u^	0.16	0.14^a^	0.13^u^	0.15	0.18	−0.01	**0.15** ^a^	0.04^v^	−0.08	0.14^a^
CHOL19	0.24^a^	0.14^u^	0.16	0.25^a^	0.12^u^	0.15	0.18	−0.02	**0.27** ^b^	−0.08^v^	−0.09	0.25^a^
FATP12	0.18^a,b^	0.43^u^	0.14	0.18^a,b^	0.42^u^	0.13	−0.03	−0.12	**0.20** ^b^	0.15^v^	−0.05	0.16^a^
FATP21	0.22^a,b^	0.52^u^	0.16	0.22^a,b^	0.53^u^	0.16	−0.09	−0.10	**0.26** ^b^	0.16^v^	−0.10	0.22^a^
GLUC8	0.12^a^	0.02^u^	0.22	0.12^a^	0.01^u^	0.20	0.02	0.06	0.11^a^	0.04^u^	−0.09	0.10^a^
GLUC19	0.01^a^	0.04^u^	0.19	0.01^a^	0.02^u^	0.18	−0.04	0.05	0.01^a^	−0.05^v^	−0.11	0.01^a^
TRGL8	0.08^a^	0.05^u^	0.16	0.09^a^	0.05^u^	0.15	0.04	0.03	0.09^a^	−0.07^v^	−0.07	0.08^a^
TRGL19	0.14^a^	0.15^u^	0.15	0.14^a^	0.15^u^	0.14	0.03	−0.05	**0.17** ^b^	0.03^v^	−0.09	0.15^a,b^

The numbers in bold (per row) show the best GEBV accuracies ($\rho_{\hat{g}\_y^{*}}$) across models, identical results between 2 or more models are underlined. Superscripts were used to identify significant differences among $\rho_{\hat{g}\_y^{*}}$ (letters a and b) and among $\rho_{\hat{t}\_y^{*}}$ (letters u and v) obtained with the different models.

a $\rho_{\hat{g}\_y^{*}}$ = correlation between additive genetic effect and corrected phenotypes; $\rho_{\hat{t}\_y^{*}}$ = correlation between gene transcripts effect and corrected phenotypes; $\rho_{\hat{g}\_\hat{t}}$ = correlation between the additive genetic and gene transcripts effects; $\rho_{\hat{g}\_\hat{gt}}$ = correlation between the additive genetic effect and the interaction between genetic and gene transcript effects; $\rho_{\hat{t}\_\hat{gt}}$ = correlation between the additive genetic effect and the interaction between genetic and gene transcript effects; $\rho_{\hat{t_{c}}\_y^{*}}\;$= correlation between gene transcripts conditioned on SNP genotypes and corrected phenotypes; $\rho_{\hat{g}\_\hat{t_{c}}}\;$= correlation between the additive genetic effect and gene transcripts conditioned on SNP genotypes.

b For a description of the traits, see Table 1.

c For a description of the models, see Table 2.

## Discussion

Here, we investigated parametric and nonparametric approaches to leverage transcriptomic data for the prediction of complex phenotypes. To accomplish that, we used 478 animals from the DO Mouse population (Svenson *et al.* 2012), for which information on phenotypes (Churchill *et al.* 2012) for a wide range of quantitative traits, SNP genotypes and gene transcript levels from liver tissue (Tyler *et al.* 2017) were available on the same animals. We used the genomic (GBLUP) and transcriptomic (TBLUP) BLUP models to evaluate the value of these omics data to predict phenotypes. In addition, we evaluated models to integrate genome and transcriptome data by modeling both layers independently (GTBLUP) or including an interaction component between the genome and transcriptome (GTIBLUP). Finally, we developed and tested the new GTCBLUP model that removes the between-omics-layer information redundancy. The GBM algorithm was investigated as a nonparametric approach potentially able to perform variable selection and capture nonlinear effects by fitting either SNP genotypes (GGBM), gene transcript levels (TGBM) or to integrate both layers implicitly modeling interactions within- and between-omics layers (GTGBM).

Using data from 6 distinct traits measured at 2 or 3 time points (resulting in 13 traits in total), we first assessed the proportion of phenotypic variance explained by each variance component included in the parametric models (Figs. 1 and 2). When using transcripts as predictors, 2 main patterns were observed. For 10 out of 13 traits (Figs. 1 and 2), the TBLUP model explained much a larger portion of the phenotypic variance than GBLUP. The observation that the portion of variance explained by gene transcripts is strongly trait-specific is in line with results observed when assessing the proportion of variance from gene transcripts for complex traits in *Drosophila* (Morgante *et al.* 2020). Ehsani *et al.* (2012) have analyzed data from an F2 mice population using models integrating genotype markers and liver transcriptomics data. The authors reported that transcripts explained 79%, while genotypes explained 36% of the phenotypic variance for body weight at 8 weeks of age. It is important to emphasize that in Ehsani *et al.* (2012), RNA samples were measured at the same time point as phenotypes were collected. On the other hand, studies have observed that genetic markers always explained a larger portion of variance than transcripts in maize (Guo *et al.* 2016; Azodi *et al.* 2019) and *Drosophila* (Li *et al.* 2019). The conflicting results found in literature may reflect the transient nature of how genes are expressed (Kleyman *et al.* 2017). Differently from genotypes, transcripts are affected by many factors such as the tissue from where samples are collected, the moment in life of sampling and the environmental conditions that the animal was exposed to. These variables most likely impact the variance explained (and concomitantly prediction performance) by transcripts.

The stability of gene transcripts over time has been a topic of interest as it might affect the genotype-to-phenotype link (Karlovich *et al.* 2009). Bryois *et al.* (2017) have analyzed transcriptomics data from whole-blood samples in unrelated human individuals collected at 2 time points, separated by 22 months. The authors reported correlations between transcriptomics data of individuals at both time points ranging from −0.50 to 1.00, with an average of 0.31 across the approximately 18,000 genes considered. This indicates that while genome-wide there is a huge variation in stability of expressions across genes, the expression of some genes is highly stable between time points as far as 22 months apart. In this study, transcripts were measured when the mice were 26 weeks of age, while all phenotypes were recorded at younger ages (from 8 to 21 weeks of age). Phenotypes recorded closer to 26 weeks of age had a larger proportion of phenotypic variance explained by transcripts than measurements made earlier in the animal’s life for the same phenotype (Figs. 1 and 2). For BW and FATP the transcripts explained a larger proportion of phenotypic variance than genotypes at all time points. For BMD, CHOL, GLUC, and TRGL, this was the case when there was 4 (BMD) to 6 weeks (CHOL, GLUC, and TRGL) in-between measuring phenotypes and transcripts, while genomics explained more phenotypic variance when this time frame increased to 14 (BMD) or 18 weeks (CHOL, GLUC, and TRGL) in-between. In Azodi *et al.* (2019), gene transcripts were quantified from whole seedlings, while phenotypes were recorded at a much older age and authors have observed limited predictive ability of transcriptomics data. Together with results from literature, our findings seem to confirm that the magnitude of the association of phenotypes with gene expressions may be time dependent. It is important to emphasize here that although this outcome could have been expected beforehand, to our knowledge it is the first time that this link between amount of variance explained by transcript vs the time difference between measuring transcripts and phenotypes has been shown empirically. The relationship between the tissue from where RNA samples are taken and the phenotype being analyzed can also be detrimental to omics model’s performance. The gene expression from whole maize seedlings considered in Azodi *et al.* (2019) showed no consistent improvement in predictive accuracy when compared with models considering only genomics data. Whole maize seedlings are probably less related to traits collected later in life than the gene expression from liver tissue available for the DO mouse dataset. It is widely known that the liver is strongly linked to many metabolic pathways (Ponsuksili *et al.* 2019), and therefore likely also especially to the BW and FATP traits used here, while the variation contained in a sample collected from whole seedlings do not reflect a specific tissue but a pool of all tissues in this organism. This could potentially mask any informative tissue-specific signals that could improve predictive accuracy for phenotypes. In general terms, it looks like although in many cases the variation in gene transcripts may capture higher proportions of variance from phenotypes than genetic markers, although this is dependent on the tissue and time of sampling, and these dependencies are likely to be trait specific.

When fitting both SNP genotypes and gene transcripts as predictors the portion of variance explained by SNP genotypes was drastically lower than for the GBLUP model. Ehsani *et al.* (2012) and Takagi *et al.* (2014) observed a reduction in captured genetic variance by SNP genotypes of around 50% when fitting genotypes together with transcripts compared to models fitting only genotypes as predictors for complex traits in other mice populations. This seems to confirm the hypothesis that there is redundant information between the genome and transcriptome layers (Wade *et al.* 2022), as also shown to be the case in *Drosophila* (Morgante *et al.* 2020). In our experience, it seems that the closer the phenotype analyzed is to the moment of RNA sampling, the higher the decrease in genetic variance captured by SNP genotypes in GTBLUP and GTIBLUP. This was observed for almost all traits we analyzed in different magnitudes. Takagi *et al.* (2014) analyzed circulating cholesterol at 10 weeks of age in mice and reported a large decrease in the genetic variance captured from SNP genotypes from models including only SNP genotypes ($g^{2}$ = 46%) and together with liver transcripts ($g^{2}$ = 19%) also measured at 10 weeks of age. In this study, we observed only a smaller decrease in genetic variance estimated when comparing GTBLUP ($g^{2}$ = 28%) and GBLUP ($g^{2}$ = 38%) for CHOL8. This seems to confirm that for the same phenotype, measurements made closer to the RNA sampling are prone to exhibit this pattern in a higher magnitude than when measured farther from the RNA sampling. This was further substantiated by the results observed for GTCBLUP. By conditioning the transcripts on the genotypes, the portion of variance explained by SNP genotypes was similar to the GBLUP model, while the variance explained by gene transcripts was much lower than estimated with TBLUP, GTBLUP, and GTIBLUP.

The formal variance partitioning achieved with the BLUP models cannot be achieved with the nonparametric GBM models. To compare the performance of GBM and BLUP in terms of explained variance we investigated the model *R*^2^ within the reference set. For the GBM models, the model *R*^2^ was almost always higher than for the BLUP models (Table 3). From our results, this pattern is recognizable for almost all traits analyzed, in which the GBM algorithm is able to capture a higher portion of variance than the parametric counterpart within the reference dataset (Table 3) but fails to outperform these models when predicting in the validation set (Table 4). The presence of noise in the data, limited size of the training set and the underlying complexity of the event being modeled are often cited as common causes of overfitting in machine learning models (Vabalas *et al.* 2019; Ying 2019). Here, we used a training dataset of approximately 286 animals and the high number of predictors in the models, coupled with the unavoidable presence of collinearity within- and between-omics layers may have caused GBM models to overfit (Shalev-Shwartz and Ben-David 2014). We have observed a big impact of hyperparameters on the predictive accuracies of the GBM models (results not shown). Having access to larger datasets could help to elucidate the magnitude of this impact for the models analyzed here since it would decrease the impact of hyperparameter definition in predictive performance, improving strength of evidence for any differences found between GBM and other models tested.

Prediction performance for phenotypes can be improved by adding transcripts in addition to genotypes in the model; however, our results suggested that the magnitude of improvement is dependent on the trait analyzed (Table 4). In line with the observed differences in variance explained by model components, TBLUP showed a better predictive ability than GBLUP for most traits except CHOL19, GLUC8, and GLUC19. In contrast, Takagi *et al.* (2014) reported higher predictive accuracies for circulating glucose and cholesterol when using liver transcripts as predictors when compared with using genotypes in a different heterogeneous mice population (Valdar *et al.* 2006). A relevant aspect is that in this study phenotypes for CHOL and GLUC were collected at 8 and 19 weeks, while RNA samples were taken at 26 weeks of age. As previously mentioned, in Takagi *et al.* (2014), RNA samples and phenotypes were collected around the same age (10 weeks). In this study, phenotypes recorded at a time point closer to the moment of transcript profiling resulted in more phenotypic variance being explained by transcripts for all 6 traits (Fig. 1), and for 5 out of 6 traits in higher predictive accuracy for TBLUP (Table 4). This was very clear for FATP and BW, where the variances explained by gene transcripts in the liver were high, which biologically makes sense. The pattern of increased variance explained and higher prediction accuracy was, however, also observed for BMD where a link to gene expression in the liver is not obvious. For 4 out of 6 traits, GBLUP also yielded higher prediction accuracy when the trait was measured at later time points, which may suggest a general time dependency of variance explained, as the association of the phenotypes with the SNPs is not affected by the moment of RNA sampling. The SNPs, however, explained considerably less variance than the transcripts, while not showing a consistent increase in variance explained over time as the transcripts did. This implies that GBLUP generally had lower power than TBLUP, and suggests that the apparent time dependency of the accuracy for GBLUP, which was less pronounced than for TBLUP, should not be overvalued given the limited size of the data used.

The TGBM model outperformed the TBLUP model for several traits, which was not the case when comparing GBLUP and GGBM. This result indicates that interactions between transcripts were more easily captured or are more relevant than between SNPs. One other hypothesis is that as transcripts are more strongly linked to phenotypes than genetic markers, transcript-by-transcript interactions are also likely to affect the phenotype more strongly than SNP-by-SNP interactions (Green *et al.* 2019), hence the former is expected to have a clearer and more detectable signal. Morgante *et al.* (2020) have used the random forest model, a nonparametric ensemble machine learning method like GBM, to predict complex phenotypes in *Drosophila* using gene transcripts as predictors but did not observe a superior predictive ability when compared with the TBLUP model. While TGBM outperformed TBLUP for 7 out of 13 traits, the GTGBM only outperformed GTBLUP or GTIBLUP in only 4 out of 13 traits. This could mean that the inclusion of SNP genotypes together with gene transcripts as predictors in the GTGBM model may have impaired the ability of GBM to capture linear and nonlinear signals from within- and between-omics layers. The exact cause remains unclear, but the size of dataset together with the substantial increase in number of predictors when switching from TGBM to GTGBM may be in the roots of it. It is likely that the GBM algorithm may require more data to be able to accurately capture all patterns from the complex relationship between omics layers underlying quantitative traits. If this is indeed the case, testing these models using a larger dataset could help to confirm this hypothesis. In Azodi *et al.* (2019), machine learning models integrating genomics and transcriptomics data were also not able to outperform single-omics models in terms of predictive accuracy for phenotypes in maize using a dataset of similarly limited size as in this study.

Although the inclusion of an interaction component in GTIBLUP captured between 9% and 26% of phenotypic variance (represented by ${gt}^{2}$ in Figs. 1 and 2) within the reference set, it hardly affected the correlations of the model components with the phenotype ($\rho_{\hat{g}\_y^{*}}$ and $\rho_{\hat{t}\_y^{*}}$), and those between the model components ($\rho_{\hat{g}\_\hat{t}}$) (Table 5). The low values observed for $\rho_{\hat{g}\_\hat{gt}}$ and $\rho_{\hat{t}\_\hat{gt}}$ in GTIBLUP also seem to suggest that the interaction component is capturing a portion of variance not directly shared with $\hat{g}$ or $\hat{t}$ components, and therefore it does not affect the relationship between other components. The correlation between $\hat{g}$ and $\hat{t}$ in GTBLUP and GTIBLUP models was always higher than the correlation between $\hat{g}$ and $\hat{t_{c}}$ in GTCBLUP across all traits analyzed. Since in the GTCBLUP model the transcript relationship matrix was conditioned on the variance of SNP genotypes ($t_{c}$), the correlation between solutions for the 2 components was expected to be closer to zero than observed for GTBLUP or GTIBLUP.

In this article, we proposed the GTCBLUP model as an alternative to integrate genome and transcriptome data for genomic prediction. There has been an increasing interest in the use of intermediate omics data in animal and plant breeding (Guo *et al.* 2016; Yang *et al.* 2017; Azodi *et al.* 2019; Morgante *et al.* 2020; Christensen *et al.* 2021; Michel *et al.* 2021), such as transcriptomics, metabolomics, or microbiome data. The inclusion of new layers of omics data into genomic prediction models could arguably help in capturing additional portions of variance not explained by genotype data, but at the same time, these layers most likely contain overlapping information, increasing collinearity between predictors. Modeling the relationship between G and T components could be an efficient way to realize the added value of integrating such omics data into genomic prediction models (Wade *et al.* 2022), but this could also be a challenge given the increase in number of parameters to be estimated. The advantage of the GTCBLUP is that as a preprocessing step it conditions the variance contained in transcripts on the variance of genotypes to minimize the amount of redundant information without having to increase model complexity. In general, the GTCBLUP model was able to produce GEBV that were at least as accurate as or slightly more accurate than the GBLUP model. The percentage of variance explained by SNP genotypes in GTCBLUP was similar to that with the GBLUP model, while it was always lower when using GTBLUP and GTIBLUP (Figs. 1 and 2). The observed reduction in additive genetic variance for GTBLUP and GTIBLUP when compared with GBLUP indicates strong redundancy in information contained in the genomic and transcriptomic layers. So, the conditioning of transcripts on SNP genotypes in GTCBLUP allowed this model to perform a more accurate variance partitioning for the additive genetic component, which consequently resulted in a more accurate estimation of GEBV (Table 5). Thus, when the aim is to predict GEBV considering both genomic and transcriptomic data, of the different models considered here, the GTCBLUP model is the most suitable.

Considering applications in practice, one limitation of the GTCBLUP as well as all other models considered in this study, is that they do not accommodate missing omics information, so all reference individuals must have genomics and transcriptomics data available. In the context of breeding programs, a situation in which all reference animals have multiple omics data available is unlikely to happen due to current high costs involved in collecting this kind of information. However, based on the observed decrease in costs of genotyping which has enabled large-scale genotyping, we may expect similar developments for the costs of transcriptomics and other intermediate phenotypes in the near future (Uzbas *et al.* 2019). At the same time, there have been some recent model developments that enable including other omics data in genomic prediction, when these other omics data are not available for all animals (Christensen *et al.* 2021; Zhao *et al.* 2022).

## Conclusion

We have assessed prediction models that incorporate genetic markers and transcriptomics data in genomic prediction of complex phenotypes in mice. The proportion of phenotypic variance explained by transcripts was almost always higher when traits were measured closer to the time of measuring gene transcripts. While GBM models explained more variance in the reference data, their predictive performance did not exceed the GBLUP models. Models including SNP genotypes and gene transcripts did not consistently outperform the best single-omics models to predict phenotypes. While TGBM model was able to outperform TBLUP, this was not the case for GTGBM compared to GTBLUP and GTIBLUP. The newly developed GTCBLUP model was able to force all phenotypic variance associated with SNP genotypes into its additive genetic component, by conditioning gene transcripts on SNP genotypes. GTCBLUP generally yielded considerably lower accuracies of phenotypic predictions than the other models including SNP genotypes and gene transcripts, but it showed the best accuracies for breeding values for most traits. We recommend using the GTBLUP model for prediction of phenotypes, while the GTCBLUP should be preferred when the aim is to estimate breeding values.

## Supplementary Material

jkac258_Supplementary_Data

## Data Availability

All data associated with this manuscript, and the code developed and used to perform analyzes described in this manuscript, can be obtained at https://doi.org/10.6084/m9.figshare.15081636.v1. All software used is publicly available. Supplemental material is available at G3 online.
