# Supplementary material for: Adding gene transcripts into genomic prediction improves accuracy and reveals sampling time dependence
Source: G3 (Bethesda). 2022 Sep 26;12(11):jkac258. doi: 10.1093/g3journal/jkac258 (PMC9635642; doi:10.1093/g3journal/jkac258)
Supplement: jkac258_Supplementary_Data [file jkac258_supplementary_data.docx]

**SUPPLEMENTARY MATERIAL - Perez *et al*. (2022)**

**Table S1.** Regression coefficient of the corrected phenotypes on prediction for validation animals from all models tested. Values closer to 1 indicate less bias.

| **Trait^1^** | **Model^2^** | | | | | | | |
| --- | --- | --- | --- | --- | --- | --- | --- | --- |
|  | **Only SNP^2^** | | **Only gene transcripts^2^** | | **SNP + gene transcripts^2^** | | | |
|  | **GBLUP** | **GGBM** | **TBLUP** | **TGBM** | **GTBLUP** | **GTIBLUP** | **GTCBLUP** | **GTGBM** |
| BMD12 | 1.16 | 1.48 | 1.52 | 1.36 | 1.47 | 1.63 | 0.88 | 1.23 |
| BMD21 | 1.47 | 1.82 | 1.67 | 1.34 | 1.65 | 1.95 | 1.13 | 1.21 |
| BW10 | 1.07 | 1.46 | 1.25 | 1.46 | 1.28 | 1.44 | 0.85 | 1.16 |
| BW15 | 0.80 | 1.82 | 1.08 | 1.44 | 1.06 | 1.17 | 0.70 | 1.12 |
| BW20 | 1.01 | 1.82 | 1.09 | 1.36 | 1.09 | 1.16 | 0.82 | 1.07 |
| CHOL8 | 1.17 | 1.78 | 0.92 | 1.31 | 1.05 | 1.06 | 0.76 | 0.79 |
| CHOL19 | 0.99 | 0.79 | 0.79 | 1.43 | 0.92 | 1.00 | 0.83 | 0.69 |
| FATP12 | 0.55 | 0.76 | 0.96 | 1.16 | 0.99 | 1.09 | 0.89 | 1.18 |
| FATP21 | 1.10 | 0.83 | 1.02 | 1.25 | 1.04 | 1.12 | 0.99 | 1.14 |
| GLUC8 | 0.38 | 0.44 | 0.29 | 1.27 | 0.31 | 0.31 | 0.68 | 0.52 |
| GLUC19 | 0.35 | 1.82 | 0.33 | 1.28 | 0.36 | 0.37 | 0.26 | 0.45 |
| TRGL8 | 0.54 | 0.61 | 0.36 | 1.83 | 0.37 | 0.36 | 0.58 | 0.86 |
| TRGL19 | 0.96 | 1.12 | 0.75 | 1.15 | 0.74 | 0.85 | 0.72 | 1.23 |
| **Mean** | 0.89 | 1.27 | 0.93 | 1.36 | 0.95 | 1.04 | 0.79 | 0.97 |
| **Min** | 0.35 | 0.44 | 0.29 | 1.15 | 0.31 | 0.31 | 0.26 | 0.45 |
| **Max** | 1.47 | 1.82 | 1.67 | 1.83 | 1.65 | 1.95 | 1.13 | 1.23 |

^1^For a description of the traits, see Table 1.

^2^For a description of the models, see Table 2.

**Table S2.** Relative root-mean squared error (RRMSE) for predictions on the validation set. Lower values indicate lower prediction error.

| **Trait^1^** | **Model^2^** | | | | | | | |
| --- | --- | --- | --- | --- | --- | --- | --- | --- |
|  | **Only SNP** | | **Only gene transcripts** | | **SNP + gene transcripts** | | | |
|  | **GBLUP** | **GGBM** | **TBLUP** | **TGBM** | **GTBLUP** | **GTIBLUP** | **GTCBLUP** | **GTGBM** |
| BMD12 | 1.00 | 1.02 | 0.99 | 1.02 | 0.98 | 0.99 | 1.01 | 0.99 |
| BMD21 | 0.99 | 1.02 | 0.96 | 0.99 | 0.95 | 0.96 | 0.98 | 0.96 |
| BW10 | 0.88 | 0.90 | 0.85 | 0.87 | 0.85 | 0.85 | 0.88 | 0.85 |
| BW15 | 0.86 | 0.86 | 0.85 | 0.85 | 0.86 | 0.86 | 0.87 | 0.86 |
| BW20 | 0.87 | 0.88 | 0.84 | 0.86 | 0.84 | 0.84 | 0.87 | 0.84 |
| CHOL8 | 0.89 | 0.90 | 0.89 | 0.91 | 0.89 | 0.89 | 0.90 | 0.87 |
| CHOL19 | 0.95 | 0.97 | 0.96 | 0.97 | 0.95 | 0.95 | 0.96 | 0.95 |
| FATP12 | 0.91 | 0.91 | 0.86 | 0.87 | 0.86 | 0.86 | 0.89 | 0.87 |
| FATP21 | 0.85 | 0.87 | 0.77 | 0.78 | 0.77 | 0.77 | 0.81 | 0.77 |
| GLUC8 | 1.15 | 1.15 | 1.17 | 1.14 | 1.15 | 1.15 | 1.15 | 1.15 |
| GLUC19 | 1.31 | 1.27 | 1.33 | 1.27 | 1.31 | 1.31 | 1.34 | 1.31 |
| TRGL8 | 1.07 | 1.09 | 1.14 | 1.14 | 1.11 | 1.10 | 1.13 | 1.10 |
| TRGL19 | 1.19 | 1.22 | 1.18 | 1.16 | 1.18 | 1.17 | 1.20 | 1.21 |
| **Mean** | 0.99 | 1.00 | 0.98 | 0.99 | 0.98 | 0.98 | 1.00 | 0.98 |
| **Min** | 0.85 | 0.86 | 0.77 | 0.78 | 0.77 | 0.77 | 0.81 | 0.77 |
| **Max** | 1.31 | 1.27 | 1.33 | 1.27 | 1.31 | 1.31 | 1.34 | 1.31 |

^1^For a description of the traits, see Table 1.

^2^For a description of the models, see Table 2.

**Table S3.** Standard error estimates (from bootstrapping) of obtained accuracies as presented in Table 4.

| **Trait^1^** | **Model^2^** | | | | | | | |
| --- | --- | --- | --- | --- | --- | --- | --- | --- |
|  | **Only SNP** | | **Only gene transcripts** | | **SNP + gene transcripts** | | | |
|  | **GBLUP** | **GGBM** | **TBLUP** | **TGBM** | **GTBLUP** | **GTIBLUP** | **GTCBLUP** | **GTGBM** |
| BMD12 | 0.07 | 0.06 | 0.07 | 0.05 | 0.07 | 0.06 | 0.08 | 0.07 |
| BMD21 | 0.07 | 0.06 | 0.06 | 0.05 | 0.06 | 0.06 | 0.07 | 0.07 |
| BW10 | 0.06 | 0.07 | 0.05 | 0.06 | 0.05 | 0.05 | 0.06 | 0.06 |
| BW15 | 0.07 | 0.07 | 0.05 | 0.06 | 0.05 | 0.05 | 0.07 | 0.06 |
| BW20 | 0.07 | 0.08 | 0.04 | 0.05 | 0.04 | 0.04 | 0.07 | 0.06 |
| CHOL8 | 0.06 | 0.06 | 0.07 | 0.06 | 0.07 | 0.07 | 0.07 | 0.07 |
| CHOL19 | 0.07 | 0.06 | 0.05 | 0.05 | 0.07 | 0.07 | 0.07 | 0.07 |
| FATP12 | 0.07 | 0.08 | 0.05 | 0.06 | 0.05 | 0.05 | 0.06 | 0.07 |
| FATP21 | 0.07 | 0.08 | 0.05 | 0.05 | 0.05 | 0.05 | 0.06 | 0.06 |
| GLUC8 | 0.07 | 0.05 | 0.07 | 0.06 | 0.07 | 0.07 | 0.07 | 0.08 |
| GLUC19 | 0.07 | 0.06 | 0.07 | 0.07 | 0.07 | 0.07 | 0.06 | 0.08 |
| TRGL8 | 0.07 | 0.06 | 0.07 | 0.06 | 0.06 | 0.07 | 0.07 | 0.06 |
| TRGL19 | 0.06 | 0.06 | 0.06 | 0.06 | 0.06 | 0.06 | 0.06 | 0.06 |

^1^For a description of the traits, see Table 1.

^2^For a description of the models, see Table 2.

**Table S4.** Standard error estimates (from bootstrapping) of correlations between model components as presented in Table 5.

| **Trait^2^** | **Model^3^** | | | | | | | | | | | |
| --- | --- | --- | --- | --- | --- | --- | --- | --- | --- | --- | --- | --- |
|  | **GTBLUP** | | | **GTIBLUP** | | | | | **GTCBLUP** | | | **GBLUP** |
|  | $\boldsymbol{\rho}_{\hat{\mathbf{g}}\boldsymbol{\_}\boldsymbol{y}^{\boldsymbol{*}}}$ | $\boldsymbol{\rho}_{\hat{\boldsymbol{t}}\boldsymbol{\_}\boldsymbol{y}^{\boldsymbol{*}}}$ | $\boldsymbol{\rho}_{\hat{\mathbf{g}}\boldsymbol{\_}\hat{\boldsymbol{t}}}$ | $\boldsymbol{\rho}_{\hat{\mathbf{g}}\boldsymbol{\_}\boldsymbol{y}^{\boldsymbol{*}}}$ | $\boldsymbol{\rho}_{\hat{\boldsymbol{t}}\boldsymbol{\_}\boldsymbol{y}^{\boldsymbol{*}}}$ | $\boldsymbol{\rho}_{\hat{\mathbf{g}}\boldsymbol{\_}\hat{\boldsymbol{t}}}$ | $\boldsymbol{\rho}_{\hat{\mathbf{g}}\boldsymbol{\_}\hat{\mathbf{g}\boldsymbol{t}}}$ | $\boldsymbol{\rho}_{\hat{\boldsymbol{t}}\boldsymbol{\_}\hat{\mathbf{g}\boldsymbol{t}}}$ | $\boldsymbol{\rho}_{\hat{\mathbf{g}}\boldsymbol{\_}\boldsymbol{y}^{\boldsymbol{*}}}$ | $\boldsymbol{\rho}_{\hat{\boldsymbol{t}_{\boldsymbol{c}}}\boldsymbol{\_}\boldsymbol{y}^{\boldsymbol{*}}}$ | $\boldsymbol{\rho}_{\hat{\mathbf{g}}\boldsymbol{\_}\hat{\boldsymbol{t}_{\boldsymbol{c}}}}$ | $\boldsymbol{\rho}_{\hat{\mathbf{g}}\boldsymbol{\_}\boldsymbol{y}^{\boldsymbol{*}}}$ |
| BMD12 | 0.06 | 0.07 | 0.07 | 0.06 | 0.07 | 0.07 | 0.06 | 0.07 | 0.06 | 0.07 | 0.08 | 0.07 |
| BMD21 | 0.06 | 0.06 | 0.06 | 0.06 | 0.07 | 0.06 | 0.07 | 0.07 | 0.07 | 0.07 | 0.08 | 0.07 |
| BW10 | 0.06 | 0.05 | 0.05 | 0.06 | 0.05 | 0.06 | 0.06 | 0.07 | 0.07 | 0.06 | 0.07 | 0.06 |
| BW15 | 0.06 | 0.05 | 0.05 | 0.06 | 0.05 | 0.06 | 0.08 | 0.08 | 0.07 | 0.07 | 0.07 | 0.07 |
| BW20 | 0.06 | 0.07 | 0.07 | 0.07 | 0.06 | 0.05 | 0.08 | 0.08 | 0.06 | 0.07 | 0.07 | 0.07 |
| CHOL8 | 0.06 | 0.07 | 0.07 | 0.07 | 0.04 | 0.07 | 0.07 | 0.08 | 0.06 | 0.06 | 0.07 | 0.06 |
| CHOL19 | 0.07 | 0.07 | 0.07 | 0.07 | 0.07 | 0.07 | 0.06 | 0.07 | 0.07 | 0.07 | 0.07 | 0.07 |
| FATP12 | 0.07 | 0.05 | 0.05 | 0.07 | 0.05 | 0.07 | 0.06 | 0.08 | 0.07 | 0.07 | 0.07 | 0.07 |
| FATP21 | 0.07 | 0.07 | 0.07 | 0.07 | 0.05 | 0.07 | 0.08 | 0.08 | 0.07 | 0.07 | 0.06 | 0.07 |
| GLUC8 | 0.07 | 0.07 | 0.07 | 0.07 | 0.07 | 0.07 | 0.07 | 0.08 | 0.07 | 0.07 | 0.06 | 0.07 |
| GLUC19 | 0.07 | 0.07 | 0.06 | 0.07 | 0.08 | 0.07 | 0.07 | 0.07 | 0.06 | 0.07 | 0.06 | 0.07 |
| TRGL8 | 0.07 | 0.07 | 0.07 | 0.07 | 0.07 | 0.08 | 0.08 | 0.07 | 0.07 | 0.07 | 0.07 | 0.07 |
| TRGL19 | 0.07 | 0.06 | 0.06 | 0.07 | 0.07 | 0.08 | 0.07 | 0.08 | 0.08 | 0.07 | 0.07 | 0.06 |

^1^$\rho_{\hat{g}\_y^{*}}$ = correlation between additive genetic effect and corrected phenotypes; $\rho_{\hat{t}\_y^{*}}$ = correlation between gene transcripts effect and corrected phenotypes; $\rho_{\hat{g}\_\hat{t}}$ = correlation between the additive genetic and gene transcripts effects; $\rho_{\hat{g}\_\hat{gt}}$ = correlation between the additive genetic effect and the interaction between genetic and gene transcript effects; $\rho_{\hat{t}\_\hat{gt}}$ = correlation between the additive genetic effect and the interaction between genetic and gene transcript effects; $\rho_{\hat{t_{c}}\_y^{*}}$= correlation between gene transcripts conditioned on SNP genotypes and corrected phenotypes; $\rho_{\hat{g}\_\hat{t_{c}}}$= correlation between the additive genetic effect and gene transcripts conditioned on SNP genotypes.

^2^For a description of the traits, see Table 1.

^3^For a description of the models, see Table 2.

**Table S5.** Posterior mean and standard deviation for variance components estimated by different models for the BW and FATP traits.

| **Trait**^1^ | **VC** | **Model**^2^ | | | | |
| --- | --- | --- | --- | --- | --- | --- |
|  |  | GBLUP | TBLUP | GTBLUP | GTIBLUP | GTCBLUP |
| BW10 | G | 0.0100 (0.0022) | - | 0.0040 (0.0012) | 0.0031 (0.0011) | 0.0102 (0.0024) |
|  | T | - | 0.0175 (0.0046) | 0.0145 (0.0031) | 0.0135 (0.0028) | 0.0038 (0.0011) |
|  | GT | - | - | - | 0.0031 (0.0012) | - |
|  | E | 0.0149 (00020) | 0.0097 (0.0023) | 0.0082 (0.0015) | 0.0064 (0.0014) | 0.0114 (0.0020) |
|  | P | 0.0249 (0.0032) | 0.0272 (0.0032) | 0.0266 (0.0023) | 0.0262 (0.0021) | 0.0255 (0.0018) |
| BW15 | G | 0.0098 (0.0022) | - | 0.0042 (0.0014) | 0.0029 (0.0010) | 0.0112 (0.0024) |
|  | T | - | 0.0207 (0.0033) | 0.0176 (0.0040) | 0.0154 (0.0028) | 0.0060 (0.0016) |
|  | GT | - | - | - | 0.0030 (0.0032) | - |
|  | E | 0.0164 (0.0019) | 0.0080 (0.0014) | 0.0070 (0.0019) | 0.0060 (0.0013) | 0.0102 (0.0022) |
|  | P | 0.0262 (0.0018) | 0.0287 (0.0025) | 0.0288 (0.0031) | 0.0273 (0.0022) | 0.0274 (0.0020) |
| BW20 | G | 0.0124 (0.0028) | - | 0.0040 (0.0010) | 0.0031 (0.0009) | 0.0143 (0.0030) |
|  | T | - | 0.0242 (0.0034) | 0.0207 (0.0031) | 0.0191 (0.0029) | 0.0079 (0.0020) |
|  | GT | - | - | - | 0.0031 (0.0009) | - |
|  | E | 0.0199 (0.0023) | 0.0082 (0.0014) | 0.0073 (0.0013) | 0.0062 (0.0012) | 0.0110 (0.0026) |
|  | P | 0.0323 (0.0022) | 0.0324 (0.0027) | 0.0320 (0.0025) | 0.0314 (0.0025) | 0.0333 (0.0024) |
| FATP12 | G | 0.1839 (0.0410) | - | 0.0908 (0.0257) | 0.0741 (0.0210) | 0.2176 (0.0415) |
|  | T | - | 0.3892 (0.0716) | 0.2835 (0.0620) | 0.2559 (0.0560) | 0.1273 (0.0352) |
|  | GT | - | - | - | 0.0634 (0.0230) | - |
|  | E | 0.3201 (0.0360) | 0.1785 (0.0333) | 0.1599 (0.0296) | 0.1302 (0.0277) | 0.1827 (0.0389) |
|  | P | 0.5040 (0.0343) | 0.5678 (0.0517) | 0.5342 (0.0457) | 0.5236 (0.0431) | 0.5276 (0.0390) |
| FATP21 | G | 0.1603 (0.0383) | - | 0.0702 (0.0192) | 0.0561 (0.0153) | 0.1870 (0.0442) |
|  | T | - | 0.4228 (0.0648) | 0.3510 (0.0599) | 0.3227 (0.0555) | 0.1361 (0.0403) |
|  | GT | - | - | - | 0.0496 (0.0158) | - |
|  | E | 0.3471 (0.0364) | 0.1426 (0.0269) | 0.1285 (0.0253) | 0.1117 (0.0240) | 0.2079 (0.0449) |
|  | P | 0.5074 (0.0339) | 0.5655 (0.0491) | 0.5496 (0.0459) | 0.5401 (0.0439) | 0.5310 (0.0405) |

VC = variance component

^1^For a description of the traits, see Table 1.

^2^For a description of the models, see Table 2.

**Table S6.** Posterior mean and standard deviation for variance components estimated by different models for the BMD, CHOL, GLUC and TRGL traits.

| **Trait**^1^ | **VC** | **Model** | | | | |
| --- | --- | --- | --- | --- | --- | --- |
|  |  | GBLUP | TBLUP | GTBLUP | GTIBLUP | GTCBLUP |
| BMD12 | G | 0.3495 (0.0751) | - | 0.2303 (0.0650) | 0.2098 (0.0489) | 0.3578 (0.0805) |
|  | T | - | 0.5205 (0.1357) | 0.2526 (0.0883) | 0.2210 (0.0876) | 0.1188 (0.0368) |
|  | GT | - | - | - | 0.1306 (0.0521) | - |
|  | E | 0.5296 (0.0619) | 0.4998 (0.0754) | 0.4437 (0.0632) | 0.3601 (0.0715) | 0.4460 (0.0715) |
|  | P | 0.8791 (0.0611) | 1.020 (0.0917) | 0.9266 (0.0716) | 0.9215 (0.0701) | 0.9225 (0.0641) |
| BMD21 | G | 0.4132 (0.0781) | - | 0.2789 (0.0707) | 0.2496 (0.0548) | 0.4413 (0.0801) |
|  | T | - | 0.6063 (0.1349) | 0.2874 (0.0893) | 0.2367 (0.0794) | 0.1361 (0.0390) |
|  | GT | - | - | - | 0.1620 (0.0325) | - |
|  | E | 0.5001 (0.0601) | 0.4577 (0.0716) | 0.3776 (0.0584) | 0.2703 (0.0611) | 0.3681 (0.0652) |
|  | P | 0.9133 (0.0639) | 1.0640 (0.0942) | 0.9439 (0.0718) | 0.9188 (0.0680) | 0.9455 (0.0657) |
| CHOL8 | G | 0.0196 (0.0045) | - | 0.0139 (0.0041) | 0.0124 (0.0035) | 0.0204 (0.0050) |
|  | T | - | 0.0227 (0.0073) | 0.0106 (0.0043) | 0.0082 (0.0035) | 0.0064 (0.0020) |
|  | GT | - | - | - | 0.0086 (0.0041) | - |
|  | E | 0.0312 (0.0037) | 0.0338 (0.0045) | 0.0286 (0.0039) | 0.0231 (0.0046) | 0.0267 (0.0043) |
|  | P | 0.0510 (0.0035) | 0.0566 (0.0049) | 0.0532 (0.0040) | 0.0523 (0.0036) | 0.0535 (0.0037) |
| CHOL19 | G | 0.0353 (0.0071) | - | 0.0251 (0.0068) | 0.0217 (0.0066) | 0.0372 (0.0075) |
|  | T | - | 0.0517 (0.0139) | 0.0201 (0.0082) | 0.0153 (0.0070) | 0.0101 (0.0029) |
|  | GT | - | - | - | 0.0154 (0.0059) | - |
|  | E | 0.0426 (0.0054) | 0.0420 (0.0073) | 0.0375 (0.0059) | 0.0276 (0.0064) | 0.0351 (0.0062) |
|  | P | 0.0780 (0.0055) | 0.0937 (0.0091) | 0.0827 (0.0064) | 0.0802 (0.0058) | 0.0825 (0.0057) |
| GLUC8 | G | 0.0102 (0.0025) | - | 0.0072 (0.0023) | 0.0061 (0.0025) | 0.0098 (0.0029) |
|  | T | - | 0.0112 (0.0034) | 0.0070 (0.0025) | 0.0057 (0.0023) | 0.0052 (0.0016) |
|  | GT | - | - | - | 0.0062 (0.0062) | - |
|  | E | 0.0244 (0.0026) | 0.0257 (0.0027) | 0.0219 (0.0027) | 0.0178 (0.0033) | 0.0210 (0.0031) |
|  | P | 0.0346 (0.0024) | 0.0369 (0.0029) | 0.0361 (0.0026) | 0.0358 (0.0026) | 0.0362 (0.00255) |
| GLUC19 | G | 0.0130 (0.0031) | - | 0.0087 (0.0026) | 0.0072 (0.0039) | 0.0121 (0.0035) |
|  | T | - | 0.0214 (0.0061) | 0.0135 (0.0047) | 0.0112 (0.0045) | 0.0120 (0.0038) |
|  | GT | - | - | - | 0.0093 (0.0093) | - |
|  | E | 0.0452 (0.0039) | 0.0402 (0.0045) | 0.0382 (0.0043) | 0.0322 (0.0053) | 0.0359 (0.0049) |
|  | P | 0.0582 (0.0039) | 0.0616 (0.0048) | 0.0605 (0.0045) | 0.0599 (0.0043) |  |
| TRGL8 | G | 0.0559 (0.0137) | - | 0.0355 (0.0112) | 0.0287 (0.0146) | 0.0566 (0.0162) |
|  | T | - | 0.0575 (0.0161) | 0.0338 (0.0115) | 0.0270 (0.0098) | 0.0187 (0.0054) |
|  | GT | - | - | - | 0.0385 (0.0385) | - |
|  | E | 0.0999 (0.0119) | 0.1078 (0.0114) | 0.0908 (0.0117) | 0.0617 (0.0152) | 0.0881 (0.0142) |
|  | P | 0.1558 (0.0107) | 0.1653 (0.0126) | 0.1601 (0.0115) | 0.1560 (0.0106) | 0.1634 (0.0113) |
| TRGL19 | G | 0.0558 (0.0133) | - | 0.0356 (0.0108) | 0.0283 (0.0176) | 0.0546 (0.0153) |
|  | T | - | 0.0868 (0.0242) | 0.0507 (0.0174) | 0.0425 (0.0162) | 0.0298 (0.0090) |
|  | GT | - | - | - | 0.0448 (0.0182) | - |
|  | E | 0.1341 (0.0132) | 0.1219 (0.0152) | 0.1128 (0.0138) | 0.0794 (0.0184) | 0.1139 (0.0161) |
|  | P | 0.1890 (0.0126) | 0.2088 (0.0172) | 0.1991 (0.0145) | 0.1950 (0.0142) | 0.1982 (0.0140) |

VC = variance component

^1^For a description of the traits, see Table 1.

^2^For a description of the models, see Table 2.
